# Probing the storage stability and sensorial characteristics of wheat and barley grasses juice

**DOI:** 10.1002/fsn3.841

**Published:** 2019-01-28

**Authors:** Aiza Qamar, Farhan Saeed, Muhammad Tahir Nadeem, Abdullah Ijaz Hussain, Muhammad Asif Khan, Bushra Niaz

**Affiliations:** ^1^ Institute of Home and Food Sciences Government College University Faisalabad Pakistan; ^2^ Department of Applied Chemistry Government College University Faisalabad Faisalabad Pakistan; ^3^ University of Agriculture Faisalabad Sub‐Campus Burewala Faisalabad Pakistan

**Keywords:** barley, cereal grass juice, physicochemical properties, sensory characteristics, wheat

## Abstract

The present research study was designed for the development of functional drinks from wheat and barley grasses followed by their physicochemical and sensorial characterization. In 1st phase, wheatgrass and barley grass juices were prepared with different concentrations and were subjected for physicochemical analysis and sensorial evaluation. Moreover, these juices were analyzed for color indices, pH, acidity, and total soluble solids (TSS) during storage study at 0, 2, 4, and 6 days. Results showed that TSS and pH of juices were 1.321, 2.8900, 3.100, and 6.225, 6.032, 6.491 for T_0_, T_1_, and T_2_, respectively, whereas a slight decreasing trend in acidity was observed during storage. Furthermore, treatments and storage (days) showed nonsignificant effect on these traits; however, storage affected significantly except for a* value for color indices. Conclusively, with respect to sensory aspects, the cereal grass juices showed satisfactory indexes of acceptability and promising marketing potential.

## 1 INTRODUCTION

In recent times, there has been growing recognition of the key role of foods and beverages in disease prevention and treatment (Ozen, Pons, & Tur, 2012). Beverages are the most active functional food category because of convenience and possibility to meet consumer demands for container contents, size, shape, and appearance, as well as ease of distribution and storage for refrigerated and shelf‐stable products. Moreover, these are an excellent delivering means for nutrients and bioactive compounds including vitamins, minerals, antioxidants, fatty acids, plant extracts, and fiber, prebiotics, and probiotics. A functional beverage is a drink product that is nonalcoholic and includes in its formulation ingredients such as herbs, vitamins, minerals, amino acids or additional raw fruit or vegetables (Kausar, Saeed, Ahmad, & Salam, 2012; Sanguansri & Augustin, 2009; Wootton‐Beard & Ryan, 2011).

Functional beverages play an important role in our everyday lives. They help keep us hydrated, prevent and help address health conditions, aid in our athletic performance or simply contribute to our overall nutritional well‐being (Sun‐Waterhouse 2011). For instance, natural extracts or juices are of momentous worth, these biochemical moieties are isolated by plants, herbs, and grasses having neutraceutical attributes for utilization in various plants having neutraceutical attributes for utilization in various food‐based products. Numerous bioactive components have been considered to be utilized as therapeutic agent and a variety of these vital components are present in wheatgrass juice (WGJ) and barley grass juice (BGJ).

**Figure 1:**
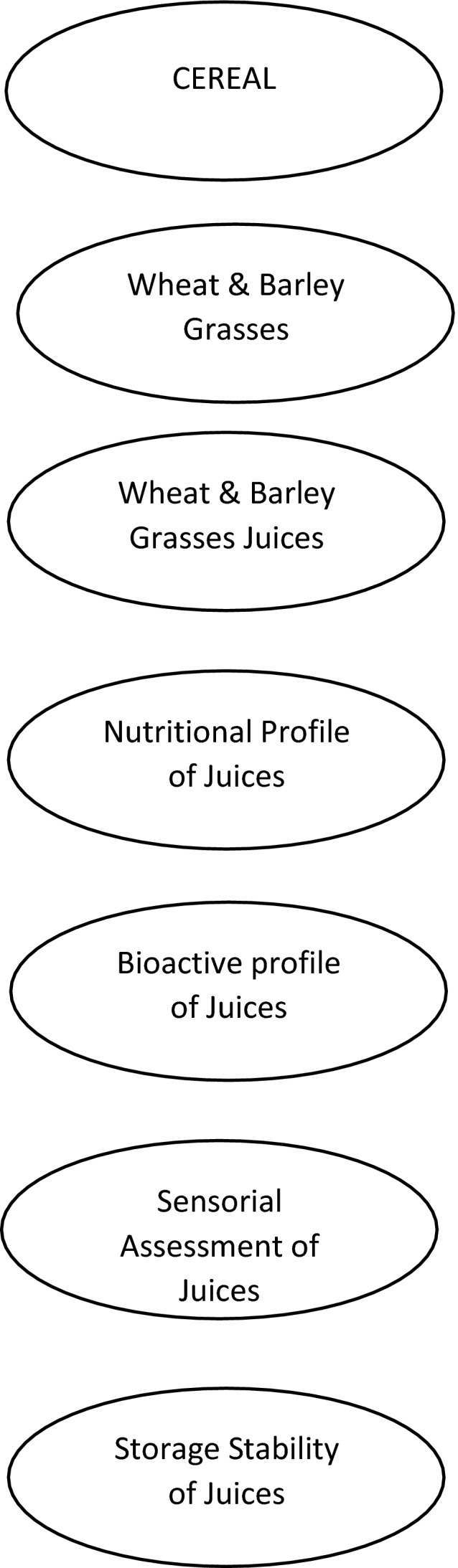
Graphical representation of whole manuscript

Green grasses juice can be made from young green leaves and dense shoots of highly nutritious grasses, which are cutoff when they have no grain. Grasses contain no gluten but grains of barley and wheat have protein which is gluten and its allergenic (Venugopal & Iyer, 2010). Cereal grass juices have high amount of chlorophyll due to which it is called as the “green blood.” Chlorophyll accounts for 70% as chemical constituents of green grasses. The most notable quality of the green grasses juice is its high chlorophyll content which is involved in regeneration of blood or acts as substitute of hemoglobin in case when deficiency of hemoglobin occurs. Lifestyle‐related disorder like anemia can be cured by the powerful effectiveness of green grasses (Padalia, Drabu, Raheja, Gupta, & Dhamija, 2010; Zeng et al., 2018). Green grass juices is one of the best magnificent drink which is involved in prevention and cure of cancer, HIV, hypercholesterolemia, diabetes owing to its strong anti‐oxidant potential (Parit, Dawkar, Tanpure, Pai, & Chougale, 2018). These are also used in detoxification of pollutants and improving the Hb level because of its blood building capabilities and protection from solar and other types of radiation, also boosting energy and immunity (Singh, Pannu, Singh, & Singh, 2010). Cereal grasses contain considerable amount of Ca, Co, Fe, Mg, K, Zn, β‐carotene, folate, pantothenic acid, vitamins B1, B2, B6, C, and E, SOD, catalase, and chlorophyll (Chand, Vishwakarma, Verma, & Kumar, 2008) (Figure 1).

Cereal grass juice contains ascorbic acid, which is seven times wealthier than an equal mass of citrus, five times better off in Fe than spinach, ten times wealthier in Ca than milk, is an important supply of vitamin cyanocobalamine, and contains 15 times as much protein as an equivalent quantity of milk. Green grasses juice is rich in vitamin K, which causes blood to clot (Rana, Kamboj, & Gandhi, 2011). Keeping in view the therapeutic role of green grasses against lifestyle‐related disorder, that is, anemia, present project was designed to prepare the functional drinks from locally grown green grasses and to analyze this product for physicochemical properties and sensorial characteristics.

## 2 MATERIALS AND METHODS

### 2.1 Procurement of raw materials

Commercially available barley and wheatgrasses were procured from Wheat Research Institute, Ayub Agriculture Research Institute (AARI), Faisalabad.

### 2.2 Sample preparation

Wheat and barley grasses were taken in a petri dish and then were placed in a hot air oven to dry the moisture content. After drying, grinding was done in the grinder with a sieve size of 6 mm. After grinding, wheat and barley grasses were again taken in a petri dish. In the end, it was placed into a hot air oven to lower the chances of air contact and humidity.

### 2.3 Product development

Fresh grass (wheat and barley) was grounded in a laboratory mortar and the juice was squeezed out through four layers of wet muslin cloth. The residue was twice resuspended in water and similarly squeezed. The filtrate was made up of the final volume with sterile water (Chin, Balunas, Chai, & Kinghonn, 2006) (Table 1).

**Table 1:** Treatments used in the study plan

Treatment	Water (ml)	Barley grass (mg)	Wheatgrass (mg)
T_0_	300		
T_1_	200	50	–
T_2_	150	100	–
T_3_	100	150	–
T_4_	200	–	50
T_5_	150	–	100
T_6_	100	–	150
T_7_	200	25	25
T_8_	150	50	50
T_9_	100	75	75

*Note*

T_0_, acts as control.

### 2.4 Physicochemical analysis

Cereal grasses juices were analyzed for the following characteristics.

#### 2.4.1 Color

The color of juices was estimated through CIE‐Lab Color Meter (CIELAB SPACE, Color Tech‐, PCM, USA). For the experiment, 5 ml of each respective juice was taken and color values like a* (−a greenness; +a redness), b* (−b blueness; +b yellowness) and L* (lightness) were recorded. The data obtained were used to compute chroma (C*) and hue angle following the method of Duangmal, Saicheuaa, and Sueeprasan (2008).

(I) $$\text{Chroma}\left(C^{*}\right)=\left[\left(a^{*}\right)2+\left(b^{*}\right)2\right]1/2$$

(II) $$\text{Hue angle (h)}=\text{tan}-1(b^{*}/a^{*})$$

#### 2.4.2 Total soluble solids

Total soluble solids (TSS) of cereal grasses juice were estimated by Hand Refractometer (TAMCO, Model No. 90021, Japan) at respective storage intervals and interpreted as per cent soluble solids (°Brix).

#### 2.4.3 pH

The representative juices were taken in 50 ml beaker and pH was recorded by pH meter (Ino Lab 720, Germany) following the method of AOAC (2006).

#### 2.4.4 Acidity

The acidity of barley and WGJs was determined during storage by adopting the guidelines of AOAC (2006). The selected sample was titrated against 0.1 N sodium hydroxide (NaOH) solution until persistent pink color.

### 2.5 Sensory evaluation of product

The cereal grass juices were rated using a 9‐point hedonic score system (9 = like extremely; 1 = dislike extremely) by trained taste panel (Meilgaard, Civille, & Carr, 2007). They were asked to express their opinion about the end product by giving a score to attributes like color, flavor, taste, texture, and overall acceptability. During the sensorial evaluation, juices with different grass concentration were placed in transparent cups, labeled with random codes. Cold water and crackers were supplied to panelists for rinsing their mouths between the samples. In each session, panelists were seated in separate booths equipped with white fluorescent lighting in an isolated room.

### 2.6 Storage study

Physicochemical attributes like L*, a*, b*, acidity, pH, chroma, hue, and TSS of the resultant cereal grass juice treatments were carried out at 0, 7, 14, and 21 days of storage according to their respective protocols as mentioned above.

### 2.7 Statistical analysis

The obtained data will be subjected to randomized design (CRD) using Statistical Package (Statistix 8.1). Levels of significance will be determined (ANOVA) using 2‐factor factorial CRD following the principles outlined by Steel, Torrie, and Dikey (1997).

## 3 RESULTS AND DISCUSSION

### 3.1 Product analysis

#### 3.1.1 Physicochemical analysis

Mean values regarding acidity, pH and TSS of different treatments of cereal grasses juices have been depicted in Table 2. These values showed that progressive increase in acidity, pH, and TSS influenced the physical characteristics significantly. The differences among all the treatments of wheatgrass, barley grass and the combination of both cereal kinds of grass juices were highly significant while chroma remained nonsignificant with exception of their momentous impact on barley grass and WGJ. Similarly, a progressive increase in hue angle, L* value, a* value, and b* value influenced the physicals characteristics of cereal grass juices significantly. The differences among all the treatments of wheatgrass, barley grass and the combination of both cereal kinds of grass juices were highly significant.

**Table 2:** Mean values for acidity, pH & TSS of cereal grass juices

Physicochemical properties of products					
Sr. No	Treatments	Acidity	PH	TSS	Chroma
1	T_0_	0.0553^c^	6.0732^ab^	1.2509^g^	239.41^a^
2	T_1_	0.1716^f^	5.8144^b^	2.5288^f^	134.27^a^
3	T_2_	0.1916^ef^	6.0494^ab^	2.7488^ef^	137.27^a^
4	T_3_	0.2116^e^	6.1094^ab^	3.0888^cde^	239.41^a^
5	T_4_	0.4333^d^	6.11871^b^	2.9958^de^	134.37^a^
6	T_5_	0.5433^c^	6.2807ab	3.4058bc	135.37^a^
7	T_6_	0.6233^b^	6.2487^ab^	4.3558^a^	137.61^a^
8	T_7_	0.5433^c^	6.3387^a^	3.2558^cd^	138.50^a^
9	T_8_	0.6233^b^	6.4287^a^	3.7358^b^	139.46^a^
10	T_9_	0.7033^a^	6.4687^a^	4.2158^a^	140.45^a^

*Notes*

Means carrying different letters are not significantly identical.

T0: Control; T1: 50 mg barley grass; T2: 100 mg barley grass; T3: 150 mg barley grass; T4: 50 mg wheatgrass; T5: 100 mg wheatgrass; T6: 150 mg wheatgrass; T7: 25 mg barley and 25 mg wheatgrass; T8: 50 mg barley and 50 mg wheatgrass; T9: 75 mg barley and 75 mg wheatgrass.

The acidity of the different treatments of cereal grass juices ranged from 0.0553 to 0.7033 where T_0_ (control) contained higher acidity value and T_9_ (combination of both kinds of grass) was lower in acidity value. A similar trend was followed by pH (5.8144 to 6.4687) and chroma (134.27 to 239.41) values whereas TSS ranged from 5.8144 to 6.4687 where T_1_ (BGJ) had lower pH value and T_9_ (combination of both grass juices). A marked increase in a*, b* value, and L* value of cereal grass juices were observed among all treatments where −0.1708 were observed in T_0_ for a* value while the lowest value was recorded for T_9_ (−1.9798). The b* content of the different treatments of BGJ and WGJ ranged from 131.35 to 140.62. Combination of barley and WGJ contained higher b* value (140.62).

The results are in accordance with Rexhepi and Renata (2015) who studied the pH values of wheatgrass, barley grass, and oat grass and stated that these values were varied from the lowest pH 3.31 for sample 2A (BGJ 30% and apple juice 70%) to 6.43 for sample no1 that is WGJ 100%, also stated that the pH of BGJ is lesser than that of WGJ.

### 3.2 Sensory evaluation of product

#### 3.2.1 Color

Mean values exhibited that the color score of juice prepared from barley grass was 5.92 ± 0.62, 6.98 ± 0.41, 7.72 ± 0.81, and 7.19 ± 0.81, respectively, for T_0_, T_1_, T_2_, and T_3_. A maximum score of color (6.85 ± 0.56) in juice prepared from wheatgrass was recorded in T_2_ and the minimum color score (5.92 ± 0.62) was observed in T_0_. Moreover, color scores of juice prepared from a combination of both barley grass and wheatgrass were observed as 5.92 ± 0.62, 5.63 ± 0.52, 6.00 ± 0.76, and 5.88 ± 0.64 for T_0_, T_1_, T_2_, and T_3_, respectively. Best juice color was observed prepared with barley grass, followed by wheatgrass and lastly a combination of both cereal kinds of grass. Level of cereal grass used (100 g) was most effective among all treatments (Table 3).

**Table 3:** Mean ± *SE* values of color

Sensory evaluation of product				
Treatment	BGJ	WGJ	WGJ+BGJ	Mean
T_0_	5.92 ± 0.62^e^	5.92 ± 0.62^e^	5.92 ± 0.62^e^	5.92^c^
T_1_	6.98 ± 0.41^bc^	6.12 ± 0.45^de^	5.63 ± 0.52^e^	6.24^b^
T_2_	7.72 ± 0.81^a^	6.85 ± 0.56^bc^	6.00 ± 0.76^de^	6.85^a^
T_3_	7.19 ± 0.81^ab^	6.40 ± 0.43 ^cd^	5.88 ± 0.64^e^	6.49^a^
Mean	6.95^a^	6.32^b^	5.85^c^	

*Notes*

Values with different letters in a column are highly significant (*p* < 0.05).

BGJ: Barley grass juice; WGJ: Wheatgrass juice; T0: Control; T1: 50 g; T2: 100 g; T3: 150 g.

#### 3.2.2 Flavor

Mean values revealed that treatment T_2_ prepared from barley grass exhibited the maximum score of flavor (7.71 ± 0.78) and the minimum flavor score (6.16 ± 0.42) was observed in T_0_. Moreover, flavor scores of juices prepared from barley grass were 7.71 ± 0.78 (T_2_) followed by 7.15 ± 0.61 (T_3_), 6.88 ± 0.45 (T_1_), and 6.16 ± 0.42 (T_0_), respectively. Furthermore, the flavor scores were recorded as, 6.16 ± 0.42, 6.43 ± 0.45, 6.98 ± 0.66, 6.48 ± 0.53 for T_0_, T_1_, T_2_, and T_3_, respectively, for juice prepared from wheatgrass (Table 4).

**Table 4:** Mean ± *SE* values of flavor

Treatment	BGJ	WGJ	WGJ+BGJ	Mean
T_0_	6.16 ± 0.42^e^	6.16 ± 0.42^e^	6.16 ± 0.42^e^	6.16^c^
T_1_	6.88 ± 0.45^bc^	6.43 ± 0.45^de^	5.73 ± 0.62^e^	6.34^b^
T_2_	7.71 ± 0.78^a^	6.98 ± 0.66^bc^	6.01 ± 0.56^de^	6.90^a^
T_3_	7.15 ± 0.61^ab^	6.48 ± 0.53^cd^	5.98 ± 0.65^e^	6.53^a^
Mean	6.97^a^	6.51^b^	5.97^c^	

*Notes*

Values with different letters in a column are highly significant (*p* < 0.05).

BGJ: Barley grass juice; WGJ: Wheatgrass juice; T0: Control; T1: 50 g; T2: 100 g; T3: 150 g.

#### 3.2.3 Sweetness

Mean values revealed that treatment T_2_ prepared from barley grass exhibited the maximum score of sweetness (7.89 ± 1.11) and the minimum sweetness score (6.01 ± 0.62) was observed in T_0_. Moreover, sweetness scores of juices prepared from barley grass were 7.89 ± 1.11 (T_2_) followed by 7.21 ± 0.66 (T_3_), 7.01 ± 0.66 (T_1_), and 6.01 ± 0.62 (T_0_), respectively. Furthermore, the sweetness scores were recorded as, 6.01 ± 0.62, 6.49 ± 0.49, 6.98 ± 0.72, 6.51 ± 0.54, and 6.01 ± 0.62, 6.11 ± 0.64, 6.41 ± 0.73, 5.99 ± 0.01 for T_0_, T_1_, T_2_, and T_3_, respectively, for juices prepared from wheatgrass and from a combination of both cereal kinds of grass (wheatgrass + barley grass) (Table 5).

**Table 5:** Mean ± *SE* values of sweetness

Treatment	BGJ	WGJ	WGJ+BGJ	Mean
T_0_	6.01 ± 0.62^e^	6.01 ± 0.62^e^	6.01 ± 0.62^e^	6.01^c^
T_1_	7.01 ± 0.66^bc^	6.49 ± 0.49^de^	6.11 ± 0.64^e^	6.53^b^
T_2_	7.89 ± 01.11^a^	6.98 ± 0.72^bc^	6.41 ± 0.73^de^	7.09^a^
T_3_	7.21 ± 0.66^ab^	6.51 ± 0.54 ^cd^	5.99 ± 0.01^e^	6.57^a^
Mean	7.03^a^	6.49^b^	6.13^c^	

*Notes*

Values with different letters in a column are highly significant (*p* < 0.05).

BGJ: Barley grass juice; WGJ: Wheatgrass juice; T0: Control; T1: 50 g; T2: 100 g; T3: 150 g.

#### 3.2.4 Sourness

Mean values exhibited that the sourness score of juice prepared from barley grass was 5.98 ± 0.63, 6.11 ± 0.64, 6.51 ± 0.75, and 6.01 ± 0.01, respectively, for T_0_, T_1_, T_2_, and T_3_. A maximum score of sourness (7.78 ± 1.11) in juice prepared from a combination of both kinds of grass (Wheatgrass + barley grass) was recorded in T_2_ and the minimum sourness score (5.98 ± 0.63) was observed in T_0_. Moreover, sourness scores of juice prepared from wheatgrass were observed as 5.98 ± 0.63, 6.12 ± 0.49, 6.89 ± 0.70, and 6.41 ± 0.54 for T_0_, T_1_, T_2_, and T_3_, respectively. Best juice was observed prepared with barley grass, followed by wheatgrass and lastly a combination of both cereal kinds of grass. Level of cereal grass used (100 g) was most effective among all treatments (Table 6).

**Table 6:** Mean ± *SE* values of sourness

Treatment	BGJ	WGJ	WGJ+BGJ	Mean
T_0_	5.98 ± 0.63^e^	5.98 ± 0.63^e^	5.98 ± 0.63^e^	5.98^c^
T_1_	6.11 ± 0.64^e^	6.12 ± 0.49^de^	6.09 ± 0.46^bc^	6.10^b^
T_2_	6.51 ± 0.75^de^	6.89 ± 0.70^bc^	7.78 ± 01.11^a^	7.06^a^
T_3_	6.01 ± 0.01^e^	6.41 ± 0.54^cd^	7.11 ± 076^ab^	6.51^a^
Mean	6.15^a^	6.35^b^	6.74^c^	

*Notes*

Values with different letters in a column are highly significant (*p* < 0.05).

BGJ: Barley grass juice; WGJ: Wheatgrass juice; T0: Control; T1: 50 g; T2: 100 g; T3: 150 g.

### 3.3 Overall acceptability

Mean values exhibited that the maximum overall acceptability score was recorded in juice prepared from barley grass was 6.15 ± 0.71, 6.78 ± 0.65, 7.69 ± 1.11, and 7.11 ± 0.62, respectively, for T_0_, T_1_, T_2_, and T_3_. A maximum score of overall acceptability (7.69 ± 1.11) in juice prepared from barley grass was recorded in T_2_ and the minimum overall acceptability score (6.15 ± 0.71) was observed in T_0_. Moreover, overall acceptability scores of juice prepared from a combination of both barley grass and wheatgrass were observed as 6.15 ± 0.71, 6.22 ± 0.51, 6.32 ± 0.73, and 5.89 ± 0.11 for T_0_, T_1_, T_2_, and T_3_, respectively. Best juice was observed prepared with barley grass, followed by wheatgrass and lastly a combination of both cereal kinds of grass. Level of cereal grass used (100 g) was most effective among all treatments (Table 7).

**Table 7:** Mean values of overall acceptability

Treatment	BGJ	WGJ	WGJ+BGJ	Mean
T_0_	6.15 ± 0.71^e^	6.15 ± 0.71^e^	6.15 ± 0.71^e^	6.15^c^
T_1_	6.78 ± 0.65^bc^	6.59 ± 0.39^de^	6.22 ± 0.51^e^	6.53^b^
T_2_	7.69 ± 01.11^a^	6.76 ± 0.72^bc^	6.32 ± 0.73^de^	6.92^a^
T_3_	7.11 ± 062^ab^	6.41 ± 0.54^cd^	5.89 ± 0.11^e^	6.47^a^
Mean	6.93^a^	6.47^b^	6.14^c^	

*Notes*

Values with different letters in a column are highly significant (*p* < 0.05).

BGJ: Barley grass juice; WGJ: Wheatgrass juice; T0: Control; T1: 50 g; T2: 100 g; T3: 150 g.

The results for sensory attributes are somehow in accordance with Rexhepi and Renata, (2015) who studied and evaluated sensory characteristics and consumer acceptance of green juices extracted from wheatgrass, barley grass, and oat grass, as well as their formulations with apple juice.

### 3.4 Storage study

Barley grass juice and WGJ were developed and analyzed for following characteristics like color indices, pH, acidity, and TSS during storage study at 0, 2, 4, and 6 days. Treatments and storage (days) showed the nonsignificant effect on these traits; however, storage affected significantly except for a* value for color indices.

#### 3.4.1 Color indices

A color indices test was done to determine the quality and consumer acceptance of the juices. Color measurement is mostly performed with the CIE‐LAB color system and its attributes are L*, a* b*, chroma and hue angle, where L*, is the indicator of lightness darkness, a* indicates greenish to reddish tonality, whereas b* represents bluish to yellowish tonality.

A gradual decrease in L* value was recorded though the changes were significant. Means squares in Table 4 (a) showed that during storage interactive effects of treatments and days ware nonsignificant while treatments were significantly affected this trait. A similar trend was followed by a* value, whereas during storage, the interactive effect of treatments was significantly affected for b* value and all the treatments were highly significant. The table indicates that the chroma and hue angle were nonsignificantly affected during the storage time period. Storage of cereal grass juices led to nonmomentous variations for hue angle and chroma.

Means regarding L* values of treatments are presented initial reading of L* values at 0 day for cereal grass juices T_0_ (control), T_1_ (BGJ) and T_2_ (WGJ) were 101.431, 103.782, and 102.672, respectively, whereas 21 days storage resulted in nonsubstantial decrease in L* value from 101.431 to 98.996 for T_0_ while for T_1_ and T_2_ were from 103.782 and 102.672 to 99.896 and 99.758, respectively. The L* value of the different varieties of cereal grasses ranged from 98.996 to 103.782 where T_1_ (BGJ) contained higher L* value and T_0_ (control) was lower in L* value.

A marked increase in a* value of cereal grass juices was observed from −0.234 in T_0_ (control) to −0.739 in T_1_ containing BGJ and −1.358 in T_2_ containing WGJ. During storage, values for a* decreased from −0.234, −0.739, and −1.358 for T_0_, T_1_, and T_2_ at the initiation of a study to −0.092, −0.593, and −1.046, respectively, at the termination of the study. Interactive effect of treatment and storage revealed that highest a* value was recorded in T_2_ (−1.358) at the beginning that decreased to −1.046 at the end of storage. Means values depicted a decreasing tendency for a* value with the passage of time from 0 days to 21st day of storage study for all treatments.

Mean values related to b* content have been symbolized in. The b* content of the different varieties of BGJ and WGJ ranged from 129.897 to 138.893. BGJ contained higher b* value ranged 138.893 and Control (T_0_) was lower in b* and ranged 129.897. Means for b* value depicted increasing trend with the passage of time at 0, 7, 14 and 21 days for T_0_ from 129.897, 131.568, 131.478, and 132.965, respectively. Furthermore, values were increased from 136.276, 136.996, 138.645, and 138.893 and 134.698, 135.459, 134.992, and 136.784 for T_1_ and T_2_, respectively, at 0, 7, 14, and 21 day of storage. A gradual increase in b* value was recorded though the changes were nonsignificant during storage.

It is obvious that chroma value increased nonsignificantly as; 136.321 was recorded in T_1_ followed by T_2_ (134.729) and T_0_ (129.992), respectively. Storage of drinks also led to nonmomentous variations for this trait. Mean values related to chroma have been symbolized in Table. The b* content of the different varieties of BGJ and WGJ ranged from 129.992 to 138.793. BGJ contained higher chroma value ranged from 138.793 and Control (T_0_) was lower and ranged 129.992.

Likewise, means for hue angle showed that storage and treatments did not affect this character significantly that were −1.678, −1.686 and −1.677 for T_0_, T_1_, and T_2_, respectively, at the initiation of the study. Means depicted that there is an increasing trend with the passage of time for hue angle for T_0_ from −1.678, −1.684, −1.694, and −1.698 at 0, 2, 4, and 6 days, respectively. Furthermore, at 0, 7, 14, and 21 day of storage values were increased from −1.686, −1.684, −1.701, and −1.712 and −1.677, −1.679, −1.689, and −1.699 for T_1_ and T_2_, respectively. The work of Mollov, Mihalev, Shikov, Yoncheva, and Karagyozov (2007) supported the present findings as they reported a decrease in L* value of beverage (Tables 8, 9, 10, 11, 12).

**Table 8:** Effect of treatments and storage on L* value of BGJ&WGJ

Storage study				
Storage intervals (days)	Treatments			Mean
	T_0_	T_1_	T_2_	
0	101.431	103.782	102.672	102.628
7	100.654	102.884	101.875	101.804
14	99.783	101.993	100.459	100.745
21	98.996	99.896	99.758	99.55
Mean	101.216	102.139	101.191	

*Note*

T_0_: Control drink; T_1_: Barley grass juice (BGJ); T_2_: Wheatgrass juice (WGJ).

**Table 9:** Effect of treatments and storage on a* value of BGJ&WGJ

Storage intervals (days)	Treatments			Mean
	T_0_	T_1_	T_2_	
0	−0.234^c^	−0.739^f^	−1.358^j^	0.777
7	−0.218^c^	−0.717^f^	−1.296^i^	0.744
14	−0.139^b^	−0.637^ef^	−1.186^h^	0.654
21	−0.092^ab^	−0.593^de^	−1.046^g^	0.577
Mean	0.1707	0.6715	1.2215	

*Notes*

Means carrying same letters do not differ significantly.

T_0_: Control drink; T_1_: Barley grass juice (BGJ); T_2_: Wheatgrass juice (WGJ).

**Table 10:** Effect of treatments and storage on b* value of BGJ&WGJ

Storage intervals (days)	Treatments			Mean
	T_0_	T_1_	T_2_	
0	129.897	136.276	134.698	133.624
7	131.568	136.996	135.459	134.674
14	131.478	138.645	134.992	135.033
21	132.965	138.893	136.784	136.214
Mean	131.477	137.702	135.483	

*Note*

T_0_: Control drink; T_1_: Barley grass juice (BGJ); T_2_: Wheatgrass juice (WGJ).

**Table 11:** Effect of treatments and storage on chroma of BGJ&WGJ

Storage intervals (days)	Treatments			Mean
	T_0_	T_1_	T_2_	
0	129.992	136.321	134.729	133.734
7	131.645	136.998	135.578	134.867
14	131.637	138.984	134.999	135.256
21	132.999	138.793	136.894	136.354
Mean	131.568	137.774	135.55	

*Note*

T_0_: Control drink; T_1_: Barley grass juice (BGJ); T_2_: Wheatgrass juice (WGJ).

**Table 12:** Effect of treatments and storage on hue angle of BGJ&WGJ

Storage intervals (days)	Treatments			Mean
	T_0_	T_1_	T_2_	
0	−1.678	−1.686	−1.677	1.680
7	−1.684	−1.684	−1.679	1.682
14	−1.694	−1.701	−1.689	1.694
21	−1.698	−1.712	−1.699	1.703
Mean	−1.6885	−1.695	1.686	

*Note*

T_0_: Control drink; T_1_: Barley grass juice (BGJ); T_2_: Wheatgrass juice (WGJ).

#### 3.4.2 pH

Mean values regarding pH value of cereal grass juices ranged from 6.62 ± 0.47% to 7.63 ± 0.53%. The results showed that the maximum pH (7.63 ± 0.53%) was found in T_2_ (WGJ) while, the minimum (6.043) was reported in T_1_ (BGJ). A marked increase in pH of cereal grass juices was observed from 6.043 in T_2_ containing BGJ to 6.181 in T_0_ and 6.396 in T_2_ containing WGJ. During storage, values for pH increased from 6.181, 6.211, 6.241, and 6.269 for T_0_ at 0, 7, 14, and 21 day of storage, similarly, the same trend was observed in T_2_ from 6.396, 6.560 at 0 and 4 day but this value decreased at the end of storage study from 6.560 to 6.460. Interactive effect of treatment and storage revealed that highest pH was recorded in T_2_ (6.560) at the 7th day of storage period that decreased to 6.550 at the end of storage. Means values depicted a decreasing tendency for pH with the passage of time from 0 to 21 days of storage study for T_1_.

The results are in accordance with Rexhepi and Renata (2015) who studied sensory attributes and consumer acceptance of cereal grass juices extracted from wheatgrass, barley grass, and oat grass and their formulations with apple juice and found that the pH values of samples vary from the lowest pH 3.31 for sample 2A (BGJ 30% and apple juice 70%) to 6.43 for sample N0.1 that is WGJ 100%, also stated that the pH of BGJ is lesser than that of WGJ. The juices involved in this research were also assessed for pH, TSS and acidity because of their direct interference in sensory attributes of juices (Table 13).

**Table 13:** Effect of treatments and storage on pH value of BGJ&WGJ

Storage intervals (days)	Treatments			Mean
	T_0_	T_1_	T_2_	
0	6.181^a^	6.043^a^	6.396^a^	6.21
7	6.211^a^	6.030^a^	6.560^a^	6.267
14	6.241^a^	6.103^a^	6.460^a^	6.268
21	6.269^a^	6.453^a^	6.550^a^	6.257
Mean	6.225	6.032	6.491	

*Notes*

Means carrying same letters do not differ significantly.

T_0_: Control drink; T_1_: Barley grass juice (BGJ); T_2_: Wheatgrass juice (WGJ).

#### 3.4.3 Acidity

Mean values regarding acidity of cereal grass juice characterization revealed that acidity ranged from 0.306 to 0622. The results showed that maximum acidity (0.622) was found in T_0_ at 0 days while, the minimum (0.306) was reported in BGJ at the start of storage study among all treatments. An increasing trend was observed in pH of cereal grass juices from 0.306 in T_1_ containing WGJ to 0.431 in T_0_ and 0.5100 in T_2_ containing WGJ. During storage, values for acidity increased from 0.431, 0.536, and 0.581 for T_0_ at 0, 7, and 14 days but a little bit increased at the 21st day of storage (0.622). Similarly, increasing trend was observed in T_2_ from 0.306, 0.390, 0.399, and 0.412 at 0, 7,14, and 21 days while maximum acidity was observed during the last day of storage for T_1_ (0.4123).

Similar trend was followed by Rexhepi and Renata (2015) who studied sensory attributes and consumer acceptance of cereal grass juices extracted from wheatgrass, barley grass and oat grass and made some treatments of wheatgrass and BGJ by adding little amount of apple juice and found that the acidity values of samples vary from the lowest 0.41 for sample 2 (BGJ 10%) to 0.23 for sample No. 1 that is WGJ 100%. Because of the direct interference of green juices with sensory attributes they assessed green juices for pH, TSS and acidity and observed that the acidity of BGJ is lighter than that of WGJ (Table 14).

**Table 14:** Effect of treatments and storage on acidity of BGJ&WGJ

Storage intervals (days)	Treatments			Mean
	T_0_	T_1_	T_2_	
0	0.431^a^	0.306^a^	0.510^a^	0.415
7	0.536^a^	0.390^b^	0.536^a^	0.482
14	0.581^a^	0.399^b^	0.550^a^	0.531
21	0.622^a^	0.412^b^	0.553^a^	0.529
Mean	0.555	0.376	0.538	

*Notes*

Means carrying same letters do not differ significantly.

T_0_: Control drink; T_1_: Barley grass juice (BGJ); T_2_: Wheatgrass juice (WGJ).

### 3.5 Total soluble solids

The mean values regarding TSS of cereal grass juices characterization revealed that TSS ranged from 1.145 to 3.510. The results showed that maximum TSS content (3.510) was found in WGJ at the 21st day while, the minimum (1.145) was reported in T_0_ (Control) at 0 days during storage study. A gradual increase was observed in TSS of T_0_ from 1.145, 1.224, 1.314 and 1.321 at 0, 7, 14 and 21 days of storage. Likewise, in T_2_ increasing trend was followed as 3.1667, 3.3667 and 3.4333 at 0, 7, 14 and 21 days. While in T_1_ TSS value increased during 21 days of storage from 2.640 to 2.890, similar increasing tendency was observed from 2.640, 2.661, 2.834, and 2.890 at 0, 7, 14, and 21st day of storage.

These results are compatible with earlier findings reported by Rexhepi and Renata (2015) who found TSS of the green juices ranged from 1 to 3.5. However, a higher percentage of TSS were found by Waghray et al. (2012) who evaluated appearance, aroma, taste and overall acceptability of WGJ for consumer acceptance to support the development of fresh juices and nutritional advantages of fresh vegetables to meet the needs of modern consumers, who increasingly buy ready to cook food or junk food to save time, without knowing that it is not a healthy diet. Carrot, wheatgrass, and bitter gourd juices were assessed for the total moisture content, total solids, TSS, and sensory analysis and analyzed that TSS of WGJ (with little addition of lemon juice) were 5.6–5.7 (Table 15).

**Table 15:** Effect of treatments and storage on TSS value of BGJ&WGJ

Storage intervals (days)	Treatments			Mean
	T_0_	T_1_	T_2_	
0	1.145^a^	2.640^a^	3.1667^a^	2.317
7	1.224^a^	2.661^a^	3.3667^a^	2.417
14	1.314^a^	2.8347^a^	3.4333^a^	2.527
21	1.321^a^	2.890^a^	3.510^a^	2.573
Mean	1.251	2.755	3.369	

*Notes*

Means carrying same letters do not differ significantly.

T_0_: Control drink; T_1_: Barley grass juice (BGJ); T_2_: Wheatgrass juice (WGJ).

## 4 CONCLUSION

Barley grass showed good hedonic response and storage stability**.** In the nutshell, utilization of these cereal grass juices in juice industry can fulfill multifarious objectives including maintaining good health of the consumer. These active ingredients also hold functional properties that are important for the juice industry. However, their contributions should be studied in order to enhance the meticulousness. Cereal grass juices should be encouraged as a functional beverage in diet based therapies against different lifestyle‐related disorders. Insufficient data are available regarding the chemical analysis of BGJ, so focus should be made and further research must be performed on this parameter.

## CONFLICT OF INTEREST

Authors declare that they have no conflict of interest.

## ETHICAL STATEMENT

This article does not contain any studies with human participants or animals performed by any of the authors. It is further certified that human and animal testing is unnecessary in this study.

## INFORMED CONSENT

For this type of study, formal consent is not required.
